# Patient Blood Management in Pregnancy

**DOI:** 10.1159/000528390

**Published:** 2023-01-06

**Authors:** Alexander Kaserer, Clara Castellucci, David Henckert, Christian Breymann, Donat R. Spahn

**Affiliations:** ^a^Institute of Anesthesiology, University and University Hospital Zurich, Zurich, Switzerland; ^b^Zentrum Gyn & Perinatal, Ärztezentrum Seefeld Hirslanden Klinik Zurich, Zurich, Switzerland

**Keywords:** Patient blood management, Pregnancy, Anemia, Iron deficiency, Postpartum hemorrhage

## Abstract

**Background:**

Patient blood management (PBM) is a multidisciplinary and patient-centered treatment approach, comprising the detection and treatment of anemia, the minimization of blood loss, and the rational use of allogeneic transfusions. Pregnancy, delivery, and the puerperium are associated with increased rates of iron deficiency and anemia, which correlates with worse maternal and fetal outcomes and places pregnant women at increased risk of obstetric hemorrhage.

**Summary:**

Early screening for iron deficiency before the onset of anemia, as well as the use of oral and intravenous iron to treat iron deficiency anemia, has been shown to be beneficial. Anemia in pregnancy and the puerperium should be treated according to a staged regimen, administering either iron alone or in combination with an *off-label* use of human recombinant erythropoietin in selected patients. This regimen should be tailored to the needs of each individual patient. Postpartum hemorrhage (PPH) accounts for up to one-third of maternal deaths in both developing and developed countries. Bleeding complications should be anticipated and blood loss reduced by interdisciplinary preventive measures and individually tailored care. It is recommended that facilities have a PPH algorithm, primarily focusing on prevention through use of uterotonics, but also incorporating early diagnosis of the cause of bleeding, optimization of hemostatic conditions, timely administration of tranexamic acid, and integration of point-of-care tests to support the guided substitution of coagulation factors, alongside standard laboratory tests. Additionally, cell salvage has proven beneficial and should be considered for various indications in obstetrics including hematologic disturbances, as well as various forms of placental disorders.

**Key Message:**

This article reviews PBM in pregnancy, delivery, and the puerperium. The concept comprises early screening and treatment of anemia and iron deficiency, a transfusion and coagulation algorithm during delivery, as well as cell salvage.

## Introduction

**Fig. 1:**
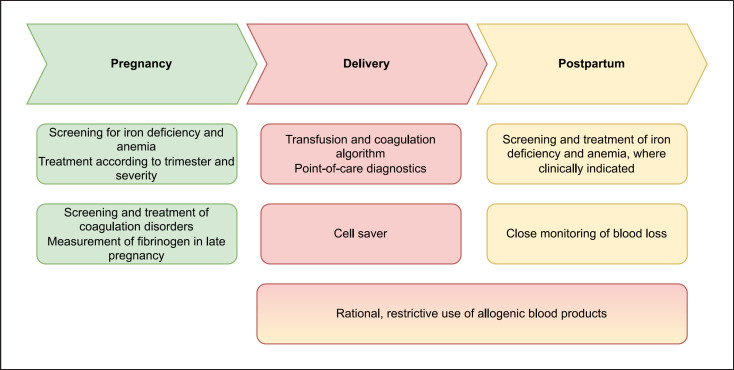
PBM in pregnancy.

Patient blood management (PBM) is a multidisciplinary and patient-centered treatment approach, comprising the detection and treatment of anemia, the minimization of blood loss, and the rational use of allogeneic transfusions. So far, the focus of PBM has been on major elective surgeries. Given the success of PBM initiatives [1], it seems urgent to extend PBM to other disciplines. Pregnant and parturient women in particular represent an important patient population in this regard (Fig. 1).

Pregnancy, delivery, and the puerperium are associated with increased rates of iron deficiency and anemia [2, 3, 4], which correlates with worse maternal and fetal outcomes. Although postpartum hemorrhage (PPH) and hypertensive complications remain the main causes of direct maternal mortality, iron deficiency anemia substantially attributes to maternal and fetal morbidity and mortality [5, 6, 7]. While treatment protocols and transfusion algorithms specifically tailored to pregnancy, delivery, and the postpartum period have led to measurable improvements in care [8], there remains room for further improvement. Meanwhile, mirroring the continued high prevalence of anemia [9] and PPH [5], the number of at-risk patients continues to rise. This article will review the state of the art on iron deficiency and anemia in pregnancy, as well as transfusion and coagulation algorithms during delivery, caesarian section, and in PPH.

## Anemia in Pregnancy and the Puerperium

Anemia in pregnancy is defined by the WHO as a hemoglobin level <110 g/L. Worldwide, up to 40% of pregnant women receive a diagnosis of anemia [10], with higher prevalence in areas of the world where access to adequate nutrition is limited. For similar reasons, special attention should be paid to the care of migrant women. In this group of patients, severe anemia is often undiagnosed and inadequately treated [11, 12]. In Europe, with relatively better access to adequate nutrition and healthcare, anemia rates during pregnancy still reach 25% [13].

The leading cause of anemia in pregnancy as well as postpartum is iron deficiency [2, 14]. The high prevalence of iron deficiency anemia is principally due to increased iron requirements resulting from increased erythropoiesis in the mother, fetal and placental consumption, and increased basal iron metabolism. In addition, many women of reproductive age are iron deficient already prior to pregnancy [15]. However, rarer causes of anemia, such as hemoglobinopathies including sickle cell anemia, should also be considered in the relevant ethnic groups.

Iron deficiency has detrimental effects on physical and mental performance, thermoregulation, immune function, neurologic function, and enzymatic function (e.g., respiratory chain function), with the consequence that maternal mortality rises with increasing severity of iron deficiency anemia [16]. In general, patients with iron deficiency anemia report symptoms of fatigue with attendant reduced ability to perform and work, reduced thermoregulation, and increased susceptibility to infection [17]. The fetal consequences of iron deficiency anemia include placental insufficiency and intrauterine growth retardation, increased preterm birth rates, cerebral developmental delay, and reduced fetal iron stores [18, 19]. Historically, a threshold ferritin concentration of 12–15 μg/L in pregnant women was considered adequate. However, newer studies, assessing iron deficiency by means of stainable reticuloendothelial iron on bone marrow specimens, have established that, in nonpregnant men and women, a serum ferritin of less than 30 μg/L correlates with significantly reduced iron stores [20, 21]. Given that a singleton pregnancy carried to term requires 500–800 mg of maternal iron [22], it would seem reasonable to adopt at least this threshold for pregnant women too. Based on this evidence, the American College of Obstetricians and Gynecologists changed their guidelines in 2009, and the British Society for Haematology theirs in 2012, adopting a ferritin of <30 μg/L as the lower limit of normal in pregnancy [23, 24]. In an unselected sample of American women in the first trimester, 14% had ferritin levels <30 ng/mL, 6% had ferritin levels <25 ng/mL, and 5% had ferritin levels <20 ng/mL [25].

## Prevention and Therapy of Iron Deficiency Anemia

The administration of iron without actual knowledge of iron status is considered controversial [26, 27]. According to the Cochrane review of Pena-Rosas et al. [27], in countries with sufficient nutritive resources there is no scientific-medical justification for preventive iron supplementation. Randomized, placebo-controlled trials do show a positive effect of iron supplementation on hematological parameters (ferritin levels, hemoglobin) and do prevent the development of anemia in pregnant women [26, 28, 29]. Nevertheless, current data do not show a positive impact on pregnancy outcome or maternal and fetal outcome [27]. The picture is, however, highly heterogenous, and preventive iron supplementation is probably still warranted in countries where there is a high pre-existing prevalence of iron deficiency. The correct dose of iron for purely preventive purposes, however, remains a matter of debate [27].

**Fig. 2:**
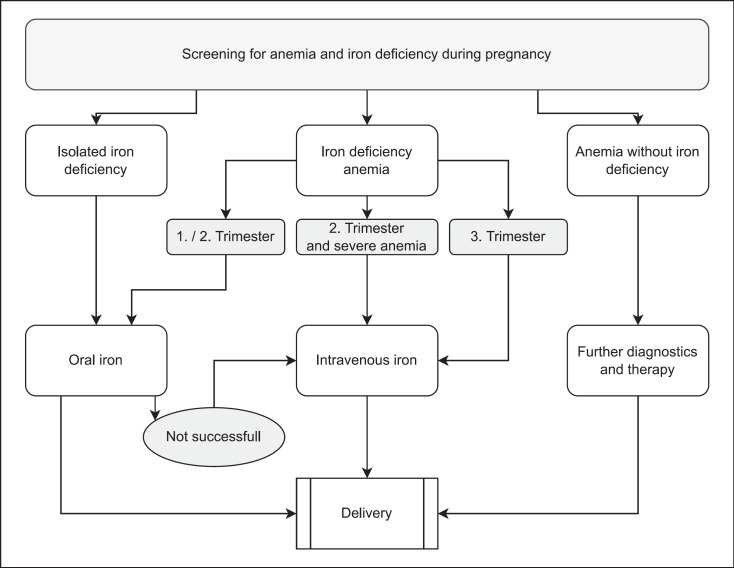
Flowchart therapy of anemia and iron deficiency in pregnancy.

By contrast, diagnosed iron deficiency should be treated, as even mild anemia can be unpredictable, suddenly worsening over the course of a pregnancy with attendant risks to the mother and fetus. The mode of therapy should take into account various factors, including time remaining until delivery, the severity of anemia, the likelihood of additional complications (e.g., preterm labor), maternal comorbidities, and the patient's wishes (e.g., refusal of allogenic blood transfusion) [30] (Fig. 2).

### Oral Iron

Oral iron is the gold standard for the treatment of mild to moderate iron deficiency anemia [31]. Higher doses, either in terms of individual dose or frequency, tend to raise circulating hepcidin levels, thereby reducing the fraction of iron absorbed in the duodenum and proximal jejunum [32]. Thus, weekly or intermittent administration (e.g., alternate-day dosing) of oral iron is recommended and has been shown to be equivalent or even superior to daily administration with fewer apparent side effects [33, 34]. The ideal dosage for intermittent or weekly administration is unclear. However, dosages between 100 and 200 mg of iron per day are a good compromise between efficacy and tolerance [30, 35]. A good response to oral iron therapy is heralded by an increase in reticulocytosis within 3–5 days, which peaks by day 8–10 after the start of therapy. Hemoglobin increases with a time lag and at approximately 2 g/L/day under best conditions or 20 g/L in 3 weeks [36]. After normalization of hemoglobin levels, oral iron should be continued for at least 4–6 months until a target ferritin level of approximately 50 μg/L and a transferrin saturation of at least 30% has been reached [30].

Gastrointestinal side effects such as constipation, heartburn, and nausea, which occur in up to 30% of patients and limit the dose which can be administered, are a major disadvantage of oral iron preparations [37, 38, 39]. One meta-analysis of 43 separate randomized studies, comparing oral iron to intravenous iron in adults (including pregnant women), reported that oral iron leads to significant gastrointestinal side effects in 70% of patients [37]. This relatively robust finding may well underlie the disappointing results of the WHO studies on reducing the prevalence of iron deficiency anemia using oral iron. As per the UK guidelines on the management of iron deficiency in pregnancy, in cases of significant side effects, some patients may benefit from either a dose reduction or a change of preparation [30].

### Intravenous Iron

Intravenous iron preparations are an important adjunct or alternative to oral iron therapy and allogenic blood transfusion. Indications for intravenous iron therapy include inadequate therapeutic response to oral iron therapy, anemia requiring rapid normalization, or simultaneous use of erythropoiesis stimulating proteins (e.g., recombinant human erythropoietin [rhEPO], NESP) [40].

Intravenous iron administration bypasses the intestinal mechanism of iron absorption and therefore often results in an increase in free iron in serum after intravenous iron administration [41]. Despite an overall benign safety profile, intravenous iron is only given from the end of the first trimester onward. This is due to theoretical concerns regarding adverse effects on the fetus during the period of embryogenesis. Reassuringly, however, it has been demonstrated that at least one formulation of intravenous iron, ferric carboxymaltose, does not cross the placenta [42]. In addition, the high molecular weight intravenous iron dextrans are notorious for high rates of severe allergic as well as nonallergic reactions have now, with the introduction of the formulations above, been phased out of clinical use [43]. However, intravenous iron supplements should only be administered when the iron status of the pregnant woman is known.

A first, hemoglobin and ferritin measurement is normally performed in the first trimester and an oral iron therapy is usually prescribed in case of hemoglobin <100 g/L and/or ferritin <30 g/L. In the case of a lack of response to oral iron (Hb levels rising by less than 10 g/L within 14 days), severe iron-deficiency anemia, intolerance of oral iron, or clinical need for rapid and efficient treatment of anemia (e.g., advanced pregnancy), intravenous iron therapy should be administered in the absence of contraindications but not before 16 weeks of gestation [44].

The intravenous iron preparations used today differ in terms of pharmacokinetics, molecular mass, toxicity, and side effects [43]. Meta-analyses comparing intravenous to oral iron in pregnancy have shown that, regardless of the preparation used, parenteral iron is more effective at increasing ferritin and hemoglobin levels up to and after delivery than oral iron [45, 46].

Several studies have confirmed the efficacy and safety of parenteral iron as ferrous saccharate in iron deficiency and anemia in pregnancy [46, 47, 48]. Indeed, data show the adverse effect profile of intravenous ferrous saccharate to be more favorable than that of oral iron and the absolute rate of serious adverse events negligible. In the various randomized controlled trials currently available, hemoglobin increases of between 13 and 25 g/L were achieved within 28 days after ferrous saccharate administration compared with 6–13 g/L with oral iron therapy [47, 48, 49, 50, 51, 52, 53, 54]. Ferric carboxymaltose was shown to be an efficacious and safe treatment in pregnant anemic women too and had even a lower adverse event rate compared to ferrous saccharate [55] and oral iron [56].

Study data are available for other intravenous iron preparations too, for example, iron polymaltose, iron gluconate, and low molecular weight iron dextrans [57]. In general, severe allergic reactions are also rare with these drugs; however due to the relatively small number of pregnant women included in these studies, these data should be interpreted with caution. Interestingly, in one study the administration of iron polymaltose appears to have a strong positive effect on quality of life in the puerperium and on iron stores in the postpartum period [58].

### Stimulation of Erythropoiesis with Recombinant Erythropoietin

The growth factor rhEPO, a glycoprotein (MG 30,400), is identical to endogenous erythropoietin and serves as a selective growth and survival factor for erythroid cells [59]. Of relevance to pregnancy, as a large protein, it does not cross the placenta [60]. It has been used clinically since 1986, primarily in patients with renal anemia who are deficient in endogenous erythropoietin [59]. In recent years, other indications have been added, including anemia of prematurity, autologous blood donation, oncology patient, and perioperative anemia therapy [61, 62, 63].

Meanwhile, there is also increasing experience using rhEPO in obstetrics [50, 64, 65]. The indications include treatment of pregnancy-related anemia associated with chronic renal failure, in cases of hypoproliferative anemia or severe anemia during pregnancy (e.g., Hb below 80 g/L), congenital hemoglobinopathies, in patients not responding to iron alone and in patients who refuse blood transfusions (e.g., Jehovah's Witness) [50, 66, 67]. Two randomized trials in pregnant women with anemia, of oral [68] or intravenous iron [66] with or without rhEPO, have established the safety of rhEPO in pregnancy for the mother and fetus. They further showed that treatment with rhEPO, rather than iron alone, leads to faster improvement in hematological indices of anemia. However, while rhEPO can shorten the interval to normalization of hemoglobin concentration in anemia, a prerequisite is that sufficient iron for erythropoiesis is available. This is most effectively provided via the parenteral route. If, in the presence of stimulation of erythropoiesis by rhEPO, sufficient iron is not available, a so-called functional iron deficiency occurs, which prevents sufficient hemoglobin synthesis [69].

According to present results, the combination of rhEPO and parenteral iron is superior to iron therapy alone in terms of raising hemoglobin concentration and may be considered as an alternative in severe anemia or rejection of allogenic blood transfusion [66]. The effect of rhEPO is dose dependent. According to our own experience, single doses of 150–300 IU/kg body weight are sufficient, although the dose may have to be repeated, depending on the response. In order to ensure an optimal cost-benefit ratio, anemia in pregnancy and the puerperium should be treated according to a staged regimen, administering either iron alone or in combination with rhEPO, depending on the cause, the response to iron supplementation, and severity of the anemia. This regimen should be tailored to of the needs of each individual patient. It is important to balance the bleeding risk against the prothrombotic risk case by case. Although the hemoglobin levels are lower during pregnancy, the hemoglobin mass is increased. With higher hemoglobin concentrations, thromboembolic events may increase. So far, no case of thromboembolism after rhEPO use in pregnancy is described. However, hemoconcentration should be avoided and the therapy should be stopped when hemoglobin levels of 100–105 g/L are reached. Higher hemoglobin levels may not be aimed at. Finally, and of note, the use of rhEPO in these contexts is limited by its “off-label” designation and also by cost.

## Physiological and Hematological Changes in Pregnancy and the Puerperium

The coagulation cascade as classically taught, with extrinsic and intrinsic pathways converging on a final common pathway of thrombin formation, does not fully capture the true nature of in vivo clot formation [70]. A more up-to-date account is the cell-based model of coagulation, which emphasizes the role of tissue factor (TF)-bearing cells and platelets and which splits coagulation into initiation, amplification, and propagation phases [71].

In this model, the exposure of TF-bearing cells to factor VII activates the extrinsic pathway leading to the formation of a small amount of thrombin. This small amount of thrombin then amplifies the pro-coagulant response by activating cofactors, factor XI, and platelets. Following that, a large burst of thrombin is formed on the platelet surface via the intrinsic pathway. Thus, the extrinsic and intrinsic pathways are not redundant but rather work in concert to bring about adequate secondary hemostasis ending in conversion of fibrinogen in fibrin monomers [72].

**Fig. 3:**
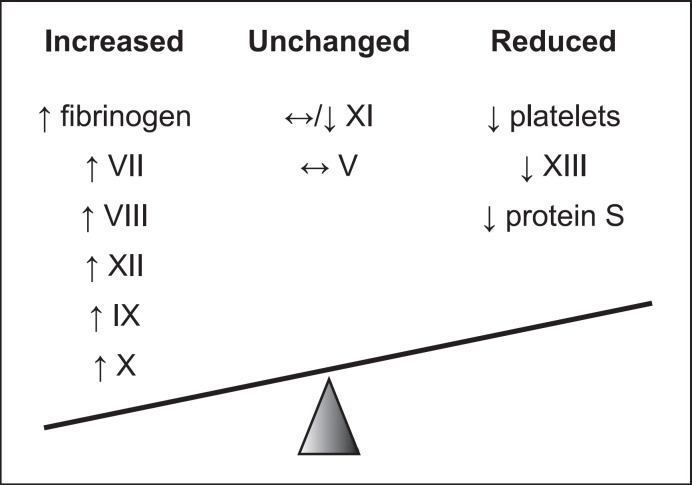
Changes of coagulation factors in pregnancy.

Pregnancy is characterized by multiple changes to this system [73] (Fig. 3). First, plasma volume increases by as much as 40%, while red blood cell volume increases by only 25%. The greater rise in plasma volume leads to a decrease in hemoglobin concentration, giving rise to what is known as the physiological anemia of pregnancy [74]. Second, as many as 8% of pregnant women also develop a thrombocytopenia, with platelet counts of less than 150,000/mm^3^, due to a combination of hemodilution, and rapid turnover in the uteroplacental unit [75, 76].

Third, and perhaps most importantly, there is an altered baseline coagulation status in pregnancy. The most notable change with respect to the nonpregnant state is a progressive rise in fibrinogen (factor I) concentration, peaking at 24 h postpartum [77, 78, 79, 80]. Factor VII [77, 78, 81], VIII [77, 78, 82], IX [77, 78, 83, 84], X [81, 82, 83, 84], and XII [77, 82, 83, 84] levels also increase up to term. The concentration of factor V appears to be unchanged [84, 85] and that of factor XI to be either unchanged or decreased [77, 78, 82, 83, 84]. Factor XIII levels show a reduction over the course of pregnancy, being lowest in the last trimester [86, 87]. In addition, intrinsic antagonists of coagulation decrease, most prominently, for example, protein S, or remain unchanged [78, 84]. This results in a hypercoagulable state, reflected in the high burden of thrombosis and thromboembolism in pregnancy and the puerperium [5, 6, 7].

Standard laboratory tests of coagulation do not reliably reflect the hypercoagulable state of pregnancy [88, 89, 90]. By contrast, multiple TEG studies have characterized the development, over the course of pregnancy, of a progressively more hypercoagulable condition [90, 91, 92, 93, 94], which persists for up to 4 weeks postpartum [95]. At term, TEG values for R and K are typically lower and for MA and α angle are higher than in the nonpregnant population [96, 97, 98, 99]. Likewise, studies using ROTEM similarly demonstrate hypercoagulability in pregnant and postpartum women. Armstrong et al. [100] found that patients in the third trimester of pregnancy have significantly lower CT, CFT and higher MCF and α angle values than the nonpregnant population across the INTEM, EXTEM, and FIBTEM assays [100]. As with TEG, these changes develop over the course of pregnancy [88] and persist for up to 3 weeks postpartum [101, 102].

## Transfusion and Coagulation Algorithm during Delivery with Focus on PPH

Coagulation disorders in the setting of preeclampsia, HELLP syndrome, or disseminated intravascular coagulation often result in high blood loss. Other, more common, situations with a risk for massive hemorrhage arise from the placenta accreta spectrum, or placental abruption. 90% of patients with placenta accreta spectrum have blood loss >2,000 mL, which is associated with an increased risk of allogeneic blood transfusion. PPH is defined as blood loss in excess of 500 mL independent of delivery mode [103].

The four leading causes of PPH are uterine atony, accounting for up to 80% of cases, as well as trauma, placental disorders, and coagulation defects, although PPH can also occur in women without any of these risk factors. These are also known as the “four Ts”: tone, trauma, tissue, and thrombin [104, 105]. As PPH accounts for up to one-third of maternal deaths in both developing and developed countries, it is of concern that its incidence is increasing. This is thought to be the result of a higher prevalence of uterine atony, increasing maternal age, use of reproductive medicine, and cesarean deliveries [106]. Despite the successful implementation of massive transfusion protocols and PBM in other medical fields, the standardization and widespread consistent application of these principles to obstetricians and midwives with focus on hemostatic management continues to lag behind other areas [107, 108].

Measures for the prevention of PPH suggested by the World Health Organization (WHO) include the administration of uterotonics, primarily oxytocin, late cord clamping (as allowed by the condition of the neonate), controlled cord traction for placental removal, and assessment of uterine tone in all women [109]. The treatment of PPH should take an interdisciplinary approach, with obstetricians treating the uterine atony and controlling bleeding in the surgical field, while anesthesiologists focus on the correction of any existing or developing coagulopathy [106]. A recent meta-analysis confirmed the use of oxytocin as the first-line uterotonic, underlining its superiority over misoprostol with a more favorable adverse-events profile and decreased blood loss [110]. In addition, as a result of the WOMAN trial, a large international, randomized, double-blind, placebo-controlled trial of over 20,000 women, tranexamic acid (TXA) has emerged as a key player in the treatment of PPH. It significantly reduces the mortality rate due to bleeding, although not the number of hysterectomies, with no increase in adverse effects. Administration should occur as soon as possible, ideally within the first 3 h of bleeding onset, with a second dose of 1 g after 30 min if bleeding persisted [111]. Interestingly, every 15 min treatment was delayed, the survival benefit decreased by 10% [112]. Should bleeding persist despite these initial preventative measures, institutions should have an escalating treatment algorithm at the ready. These further steps require the involvement of multidisciplinary teams and can include switching to prostaglandins, uterine balloon tamponade, further pelvic artery embolization, or hysterectomy as a last option to control the bleeding [113].

Considering the importance of rapid correction of hemostasis during PPH, viscoelastic point-of-care tests, such as ROTEM and TEG, have been proven to be of great value in the assessment of bleeding, by allowing for individualized coagulation management according to each patient's needs [114]. PPH algorithms that include thromboelastometry reduce the rate of blood product transfusion as well as transfusion-related reactions and lower costs [115]. Furthermore, low fibrinogen levels, which can be detected by ROTEM's FIBTEM channel, correlate with severity of PPH [116]. Fibrinogen plays a key role in hemostasis and is essential for clot formation. During major bleeding, fibrinogen is the first coagulation factor falling to a critically low level [117]. Therefore, fibrinogen is a key target for the initial treatment of bleeding. However, randomized controlled trials questioned the benefit of early fibrinogen administration by showing no clear superiority in regard to containing blood loss or reducing transfusion requirements [118, 119, 120]. This might be explained by the fact that fibrinogen is increased during pregnancy. Thus, there is a greater delay until fibrinogen reaches critically low levels in PPH. Regarding factor XIII, higher prepartum levels may correlate with a reduced risk of PPH, proposing timely analysis and substitution for PPH prevention [121]. In summary, it is recommended that facilities have a PPH algorithm, which incorporates early diagnosis of the cause of bleeding, optimization of hemostatic conditions, timely administration of TXA and oxytocin, and integration of point-of-care tests to support the guided substitution of coagulation factors, alongside standard laboratory tests [106].

## Cell Salvage

Focusing on the second pillar of PBM, the reduction of perioperative red blood cell loss, the use of a cell salvage can also be applied in a peripartum setting. Historically, the idea of collecting a patient's own blood was born in obstetrics when the case of a woman who died of PPH provoked William Highmore to write a letter to The Lancet in 1874, stating that re-transfusing the lost blood could have saved the patient's life. In the past few decades, cell salvage has been implemented in various surgical settings. Despite this auspicious start, clinicians were initially cautious in obstetrics, due to concerns regarding amniotic fluid embolism and the re-transfusion of fetal debris potentially causing maternal alloimmunization. However, a focused review by Goucher et al. [122] of over seven studies including several 100 patients found no evidence of severe complications [122]. Nonetheless, in the obstetric setting specific safety measures are recommended, including the use of a separate suction source for amniotic fluid, only starting blood collection after delivery of the placenta [123]. Additionally, using a leukocyte depletion filter can further reduce the transfusion of amniotic fluid markers and bacteria [124, 125]. Studies show that when collected blood is filtered through a leukocyte depletion filter, fetal squamous cells are present in levels comparable to those in maternal blood after the placenta is separated [123, 126] and that amniotic-fluid-derived TF, which can cause disseminated intravascular coagulation, can be successfully removed [123].

Within obstetrics, cell salvage should be considered for various indications, especially various forms of placental disorders [127]. Further, the use of cell salvage can be considered for women with an increased risk of blood loss or transfusion requirements due to underlying hematologic disturbances [122, 128]. Cell salvage allows for rapid autologous transfusion lowering the use of allogenic red blood cell transfusion. This is of particular importance in women requiring hysterectomy after cesarian delivery due to placenta accreta [124, 127]. A recently published meta-analysis with over 5,800 patients highlighted additional benefits of cell salvage, such as decreased transfusion-related adverse events and shorter hospital stay [129]. In regard to economic value, studies vary on the cost-benefit of cell salvage as compared to packed red blood cell units [130, 131]. Given this, it is important to remember that cell salvage can also be used for collection of blood only, such that if an insufficient amount of blood is collected, the expensive wash phase prior to re-transfusion does not need to be carried out. It should be noted that these results depend on hospital-specific and patient-specific factors and so cannot be applied to every clinic and should be individually assessed based on availability and caseload [132]. In summary, the advantages of using cell salvage system in cesarian deliveries with a high risk of bleeding in a patient group with a high incidence of anemia should not be underestimated. Just as it has proven beneficial in other surgical settings, so too does cell salvage have a place in the care of high-risk obstetric patients [133].

## Conclusion

Pregnancy, delivery, and the puerperium are associated with increased rates of iron deficiency and anemia, which correlates with worse maternal and fetal outcomes and places pregnant women at increased risk of obstetric hemorrhage. Iron deficiency and anemia in pregnancy and the puerperium should be treated according to a staged regimen, administering either iron alone or in combination with human recombinant erythropoietin in selected patients. This regimen should be tailored to of the needs of each individual woman. It is recommended that obstetric facilities have a PPH algorithm, which incorporates early diagnosis of the cause of bleeding, optimization of hemostatic conditions, timely administration of TXA as well as oxytocin, and integration of point-of-care tests to support the guided substitution of coagulation factors, alongside standard laboratory tests. Cell salvage has been shown to be safe and beneficial and should be considered for various indications in obstetrics.

## Conflict of Interest Statement

David Hencker and Clara Castellucci have no conflicts of interest to declare. Alexander Kaserer received honoraria for lecturing from Bayer AG, Zürich, Switzerland. Christian Breymann is medical advisor in the field of iron therapy in OBGYN and received honoraria for lecturing from Vifor International and Pierre Fabre Switzerland. Donat R. Spahn's academic department is receiving grant support from the Swiss National Science Foundation, Berne, Switzerland, the Swiss Society of Anesthesiology and Perioperative Medicine (SSAPM), Berne, Switzerland, the Swiss Foundation for Anesthesia Research, Zurich, Switzerland, Vifor SA, Villars-sur-Glâne, Switzerland, and Vifor (International) AG, St. Gallen, Switzerland. Dr. Spahn is co-chair of the ABC-Trauma Faculty, sponsored by unrestricted educational grants from Novo Nordisk Health Care AG, Zurich, Switzerland, CSL Behring GmbH, Marburg, Germany, LFB Biomédicaments, Courtaboeuf Cedex, France, and Octapharma AG, Lachen, Switzerland. Dr. Spahn received honoraria/travel support for consulting or lecturing from Danube University of Krems, Austria, European Society of Anesthesiology and Intensive Care, Brussels, BE, Korean Society for Patient Blood Management, Seoul, Korea, Korean Society of Anesthesiologists, Seoul, Korea, Network for the Advancement of Patient Blood Management, Haemostasis and Thrombosis, Paris, France, Alexion Pharmaceuticals Inc., Boston, MA, AstraZeneca AG, Baar, Switzerland, Bayer AG, Zürich, Switzerland, B. Braun Melsungen AG, Melsungen, Germany, CSL Behring GmbH, Hattersheim am Main, Germany and Berne, Switzerland, Celgene International II Sàrl, Couvet, Switzerland, Daiichi Sankyo AG, Thalwil, Switzerland, Haemonetics, Braintree, MA, USA, Instrumentation Laboratory (Werfen), Bedford, MA, USA, LFB Biomédicaments, Courtaboeuf Cedex, France, Merck Sharp & Dohme, Kenilworth, NJ, USA, Novo Nordisk Health Care AG, Zurich, Switzerland, PAION Deutschland GmbH, Aachen, Germany, Pharmacosmos A/S, Holbaek, Denmark, Pfizer AG, Zürich, Switzerland, Pierre Fabre Pharma, Alschwil, Switzerland, Portola Schweiz GmbH, Aarau, Switzerland, Roche Diagnostics International Ltd, Reinach, Switzerland, Sarstedt AG & Co., Sevelen, Switzerland and Nümbrecht, Germany, Shire Switzerland GmbH, Zug, Switzerland, Takeda, Glattpark, Switzerland, Tem International GmbH, Munich, Germany, Vifor Pharma, Munich, Germany, Neuilly sur Seine, France and Villars-sur-Glâne, Switzerland, Vifor (International) AG, St. Gallen, Switzerland, and Zuellig Pharma Holdings, Singapore, Singapore.

## Author Contributions

Concept and design: Donat R. Spahn and Alexander Kaserer. Drafting of the manuscript: Alexander Kaserer, Clara Castellucci, David Henckert, and Christian Breymann. Figures: Alexander Kaserer and David Henckert. All authors critically edited the manuscript, approved the final version to be submitted, and agree to be accountable for the accuracy and integrity of the work.
